# Associations of Yoga as a Mind–Body Exercise and Its Components with Spiritual and Subjective Well-Being: Cross-Sectional Evidence for Potential Distress Prevention

**DOI:** 10.3390/sports14010013

**Published:** 2026-01-04

**Authors:** Orsolya Cseh, Vera Klier, István Karsai, Henriett Nagy, Gusztáv József Tornóczky

**Affiliations:** 1 Doctoral School of Psychology, Faculty of Pedagogy and Psychology, ELTE Eötvös Loránd University, 1064 Budapest, Hungary; csopabb@student.elte.hu; 2 Doctoral School of Education, Faculty of Pedagogy and Psychology, ELTE Eötvös Loránd University, 1075 Budapest, Hungary; geist.vera@tok.elte.hu; 3 Physical Education and Exercise Centre, Medical School, University of Pécs, 7624 Pécs, Hungary; istvan.karsai@aok.pte.hu; 4 Department of Personality and Health Psychology, Faculty of Pedagogy and Psychology, ELTE Eötvös Loránd University, 1064 Budapest, Hungary; nagy.henriett@ppk.elte.hu; 5 Institute of Health Promotion and Sport Sciences, Faculty of Education and Psychology, ELTE Eötvös Loránd University, 1117 Budapest, Hungary

**Keywords:** spiritual well-being, subjective well-being, yoga, yoga components, mental health

## Abstract

Yoga is increasingly practiced worldwide and is associated with diverse physical and mental health benefits, yet its spiritual dimensions remain underexplored in empirical research. This study investigated the relationship between the weekly frequency of practicing specific yoga components and levels of spiritual well-being and subjective well-being (SuWB). A total of 335 Hungarian adults (mean age = 47.2 ± 10.5 years) with an average of 10.2 ± 7.9 years of yoga experience completed a national online survey. Spiritual well-being (SpWB) was measured using the Spiritual Health and Life-Orientation Measure (SHALOM), and SuWB was assessed with the WHO-5 Well-Being Index. Statistical analyses included ANCOVA, Kruskal–Wallis H tests with post hoc Mann–Whitney U tests, and Kendall’s tau correlations. All four yoga components (āsanas, prāṇāyāma, relaxation, meditation) showed medium-sized positive main effects on SpWB (*p* < 0.001; η^2^ = 0.06–0.09) and small but significant effects on SuWB (*p* = 0.003–0.05; η^2^ = 0.03–0.04). The strongest effects were observed in the Personal and Transcendental dimensions of spirituality. Weak to moderate positive correlations were found between SuWB and the four SHALOM factors as well as the total SHALOM score. These findings indicate that regular engagement in diverse yoga practices is associated with higher levels of both spiritual connection and mental well-being, highlighting that yoga practice is associated with higher levels of spiritual connection and mental well-being, and may be relevant for mental health promotion.

## 1. Introduction

Yoga as a form of physical exercise is becoming increasingly popular in Western Societies, primarily for the purpose of maintaining and improving health [1]. However, yoga, as a traditional and complex method incorporates physical, mental, and spiritual elements as well. The various components of yoga, such as āsanas (yoga poses), prāṇāyāma (breathing exercises), relaxation, and meditation, not only affect physical well-being [2,3,4,5], but also mental and emotional health [6,7,8,9].

Yoga as a holistic mind–body method [10,11,12] integrates physical movement, breath control, and meditation to promote comprehensive health and well-being [13]. Scientific research supports the benefits of yoga, showing it can activate the parasympathetic nervous system and improve emotional regulation [14,15], with positive effects across multiple domains, including stress management, anxiety reduction, and potential improvements in neurological conditions [16,17,18,19,20,21]. Regular engagement in yoga practices has been found to support stress management even in high pressure settings [22], with very few or no side effects [23]. Among younger populations, such as children and adolescents, regular yoga practice assists in alleviating symptoms of anxiety and, to a lesser degree, depression [17]. Clinical studies among breast cancer patients have also found significant reductions in distress symptoms associated with yoga practice [24,25,26]. Systematic reviews and clinical trials investigating mind–body-oriented yoga interventions consistently indicate that yoga effectively alleviates depressive symptoms [27,28,29]. Hatha yoga has demonstrated clinically significant improvements both as an effective monotherapy for individuals with mild-to-moderate major depressive disorder [28] and as an adjunctive treatment for patients in partial remission [29]. Based on the studies cited above, yoga has been shown to positively contribute to the alleviation of distress symptoms across various populations, including different age groups and health statuses, further supporting its relevance to adult practitioners.

Numerous empirical studies confirm yoga’s benefits on both physical and mental health. These positive effects include improved physical fitness, alleviation of various health problems (from injuries to chronic diseases), and improvements in mental health indicators such as mindfulness, resilience, happiness, and overall well-being [8].

Subjective well-being (SuWB), a primary indicator of mental health, encompasses self-acceptance, personal growth, a clear life purpose, positive relationships, effective environmental mastery, and autonomy. These elements closely align with the traditional Yogic worldview. Yoga practice is widely acknowledged for its significant positive impact on SuWB [30]. Research indicates that regular engagement in yoga is associated with a range of psychological benefits, including enhanced healthy psychological functioning, increased positive affectivity, and greater satisfaction with life, alongside a reduction in negative affect [31]. Yoga interventions have demonstrated effectiveness in alleviating symptoms of depression, anxiety, distress, and stress, thus contributing to overall improved mental health [31,32].

This positive influence on mental well-being is often attributed to yoga’s role as a relaxation practice [33], its ability to promote self-knowledge, and its contribution to self-regulation and resilience [34]. Moreover, yoga has been found to decrease perceived stress and improve mindfulness traits, which are crucial for psychological health [35]. Some studies suggest that the integration of spiritual and ethical components within yoga practice, beyond mere physical exercise, may offer additional psychological advantages. These findings collectively underscore yoga’s potential as a valuable tool for fostering psychological resources and mental health promotion [31].

The spiritual aspect of yoga is frequently overlooked or insufficiently represented in Western scientific studies [36,37,38]. In traditional yoga philosophy, spirituality is related to the “soul” or “spirit,” the innermost core of a person, which is interpreted as pure consciousness. The goal of spiritual yoga practices is to connect with the self (atma) and with God (Paramatma). Furthermore, the literature shows that practising yoga promotes a sense of inner peace [39,40]. Yoga practice particularly develops resilience in times of collective stress and strengthens both religious/spiritual and subjective well-being [41].

Spiritual well-being (SpWB) encompasses personal, communal, environmental, and transcendental dimensions [42,43]. These dimensions contribute to an individual’s overall health and quality of life, and can be effectively measured, for example, using the Spiritual Health and Life-Orientation Measure (SHALOM) tool [44]. Review by Csala et al. [1] has shown that yoga practice is positively associated with various dimensions of spirituality, including increased spiritual pursuit, the search for inner wisdom, the development of an integrative worldview, and a sense of meaning and peace, faith, hope, compassion, and happiness. Regular practice is key here; many practitioners start with physical motivations, and move toward spiritual development over time.

While the benefits of yoga on well-being are increasingly acknowledged, its distinctions from conventional physical activities, such as sports, remain underexplored. Both yoga and sports are highly beneficial for well-being, by developing strength, endurance, flexibility, balance, and mental focus. While yoga and sports pursue common objectives, they offer distinct yet equally valuable approaches that improve overall health [45].

Although yoga is not categorised as a sport, its inclusion in the field and its examination within sport sciences are warranted. Just as sports training primarily drives physical activity, the āsana component of yoga (the central focus of Western yoga practice) also comprises physical exercises that closely resemble elements of gymnastics, including poses with moderate- to vigorous-intensity activity [46]. Sports training typically aims to develop the muscular, skeletal, cardiovascular, and nervous systems, processes particularly critical during childhood and youth [47]. In adulthood, sports enhance aerobic work capacity, muscle strength, and functional health, actively preventing or alleviating numerous diseases such as cardiovascular issues, metabolic syndrome, and Type-2 diabetes [48,49]. Sports also aid in psychosocial development, fostering self-esteem, alleviating depression, and building social connections and a sense of belonging, which can lead to higher physical activity in later life [48,50,51].

However, competitive aspects can pose risks of injury, overtraining, and increased stress [52], and previous research on exercise addiction by Dinardi et al. [53], has also indicated that exercise itself may become a potential source of stress. Within this context, the study of yoga assumes particular relevance, as yoga practice improves core components of physical fitness such as strength, balance, and range of motion [54]. Yoga’s relevance in the field of exercise is further supported by an expanding body of sport-science literature that regularly reports on the physical and psychological effects of yoga in both athletic and non-athletic populations [46,51,55,56,57,58].

Yoga, in contrast, adopts a holistic approach, integrating breathing techniques, postures, and muscle group engagement, improving flexibility, balance, and body awareness (proprioception) [54]. Psychologically, yoga is highly effective in reducing stress, anxiety, and PTSD symptoms, while also improving self-awareness, and mental concentration [59]. It has significant physical health benefits as well, such as injury rehabilitation, improving joint mobility and managing pain [59,60,61,62]. Given their respective benefits, yoga is increasingly recognised as a valuable complement to traditional sports training as an enhancement for athletic performance by boosting flexibility, strength, balance, and body awareness [59,60,61,62] and improving emotional regulation through breathing exercises and mindfulness [59,60,63,64]. Together, sports and yoga offer a comprehensive pathway to optimal physical and mental health.

While the positive effects of yoga are well-documented, the research landscape remains fragmented. Most studies fail to capture the full complexity of yoga practice, particularly its philosophical, meditative, and spiritual components [65]. Western research often reduces yoga to mere physical exercises, overlooking its deeper spiritual and holistic aspects, and research linking yoga to mental health often lacks methodological rigour [66,67].

An important conceptual distinction must be made between SpWB and “positive mental health” (PMH) [68]. As mentioned earlier, while PMH includes psychological aspects like well-being, life satisfaction, and mindfulness, SpWB encompasses unique dimensions such as inner peace, transcendence, and faith [44]. Hendriks et al. [8] conducted a systematic review on the effects of yoga on positive mental health (PMH) (including aspects such as SuWB, life satisfaction, social relationships, and mindfulness) in non-clinical adult populations. They found only weak evidence that yoga consistently improves PMH. In contrast, Csala et al. [1] highlighted that “spiritual well-being” includes unique and distinct elements that extend beyond general mental well-being, despite some areas of overlap.

Both Csala et al. [1] and Hendriks et al. [8] drew attention to significant methodological problems in previous research. There is a high risk of bias and heterogeneity between studies:

- First yoga is treated as a single intervention, rather than examining its individual components;
- Interpreting Hatha yoga as only a physical practice, ignoring the contribution of elements such as breathing exercises and meditation;
- Overreliance on cross-sectional research designs, limited generalizability, and low specificity in measuring yoga elements.

While this study employs a cross-sectional design and therefore cannot address all methodological challenges, this study contributes to addressing these gaps through the application of Fisher’s [42] four-dimension model of the SHALOM questionnaire for comprehensive data collection and analysis. The primary objective is to systematically examine the associations between yoga practice and multiple dimensions of SpWB. Specifically, the study investigates how the weekly frequency of practicing individual yoga components—āsanas, prāṇāyāma, relaxation, and meditation—relates to the four domains of SpWB (Personal, Communal, Environmental, and Transcendental) as measured by SHALOM. In addition, it explores the relationship between SpWB and SuWB among current yoga practitioners across all Hungarian counties. By isolating the different elements of yoga and assessing their individual contributions, this research aims to provide a nuanced understanding of how yoga supports mental and spiritual health, offering insights to guide targeted, evidence-based interventions in the future.

In line with the specified research objectives, the following hypotheses were formulated:

**H1:**  *We hypothesize that higher weekly engagement with each component of yoga is positively associated with higher levels of SpWB, including its dimensions, and improved SuWB.*

**H2:**  *The four dimensions and general factors of spiritual well-being (SpWB/SHALOM) are positively associated with subjective well-being (SuWB).*

## 2. Materials and Methods

### 2.1. Procedure and Participants

A cross-sectional national online survey was conducted between 15 March and 29 April 2022, targeting adult yoga practitioners in Hungary. To be included in the sample, participants were required to be over 18 years of age and currently practicing yoga. The Hungarian Association of Yoga Teachers (HAYE) facilitated participant recruitment by supporting the dissemination of the questionnaire. Following ethical approval, all registered instructors in the HAYE database (*n* = 295) were invited via email to share the survey link with their students, thereby initiating the recruitment process. Recruitment subsequently continued through a snowball sampling approach until the conclusion of the data collection period. No predetermined sample size was set, and data collection continued iteratively until the temporal limit was reached. Participants were recruited from across all Hungarian counties, ensuring broad geographic coverage; however, the sample is not representative. The survey was administered anonymously using the Google Forms platform, with informed consent obtained prior to participation. To preserve anonymity, no technical restrictions were applied to prevent multiple submissions. Ethical approval for this study was granted by the ELTE Faculty of Education and Psychology (reference number: 2022/145).

A total of 335 current yoga practitioners were surveyed for the study. The demographic profile of the participants showed that a significant majority, 88.1% were female. Participants’ ages ranged from 18 to 78 years, with an average age of 47.2 ± 10.5 years. On average, participants had practiced yoga for 10.2 ± 7.9 years. In terms of educational background, the participants showed a high level of attainment, the majority, 69.0%, held a university degree, while 26.8% had a high school diploma, 3.0% had a PhD and a smaller portion, 1.2% had a general or skilled worker qualification. Participants from the entire country were included in the data collection. While geographically all Hungarian counties were represented, the largest concentration of participants, 59.7%, hailed from Pest county. A small segment, 4.8% (16 individuals), were listed as being from a foreign county. In terms of settlement type, the participants were distributed across various urban and rural settings: 38.8% resided in the capital city, Budapest, 7.8% lived in small towns.

### 2.2. Measures

To test our hypotheses, we employed questionnaires with robust psychometric properties that are widely utilized in international research. Spiritual well-being was assessed using the multifactorial Spiritual Health and Life-Orientation Measure (SHALOM) [69] whereas subjective well-being was measured with the WHO-5 Well-Being Index (WBI-5) which has been validated in both clinical and non-clinical populations and is commonly used for monitoring depressive symptoms [70].

#### 2.2.1. Sociodemographic and Yoga Practice Assessment

The questionnaire’s sociodemographic section collected information on participants’ gender, age, educational attainment, marital status, and county of residence. This was followed by questions related to yoga practice habits, including the duration of yoga experience and the weekly frequency of practicing yoga components such as āsanas, prāṇāyāma, relaxation, and meditation. Frequency options ranged from once a week, 2–3 times per week, 4–5 times per week, and daily. Participants were also asked to indicate whether they were yoga instructors or practitioners.

#### 2.2.2. Spiritual Health and Life-Orientation Measure (SHALOM)

The Spiritual Health and Life-Orientation Measure (SHALOM) [44], and its validated Hungarian adaptation [43], is a 20-item self-report instrument designed to assess SpWB across four core domains: Personal (connection with self), Communal (connection with others), Environmental (connection with the natural world), and Transcendental (connection with a higher power or God). Each domain is represented by five items. Participants respond to each item twice on a 5-point Likert scale, once evaluating its ideal significance for optimal SpWB and once indicating the extent to which it reflects their actual lived experience. Response options range from 1 (Very low) to 5 (Very high). SHALOM has demonstrated strong psychometric properties, with previous studies supporting its reliability and construct validity [71]. The Hungarian version was best represented by a bifactor structure, including four specific domain factors and a general spirituality factor [42]. In the current study, internal consistency was good across both Ideal and Experienced subscales: SHALOM Ideal total α = 0.91 (Personal α = 0.79, Communal α = 0.77, Environmental α = 0.79, Transcendental α = 0.96); SHALOM Experienced total α = 0.93 (Personal α = 0.87, Communal α = 0.83, Environmental α = 0.85, Transcendental α = 0.96).

#### 2.2.3. WHO-5 Well-Being Index (WBI-5)

The abbreviated version of the WHO Well-Being Index (WBI-5) [72] is a concise, 5-item instrument designed to evaluate SuWB over the preceding two-week period. Participants indicate how well each statement describes their recent experience using a 4-point Likert scale ranging from 0 (Not at all true) to 3 (Completely true). Total scores reflect overall SuWB, with higher values indicating better mental health status. The Hungarian adaptation [73] has shown strong internal consistency (Cronbach’s α = 0.85) and supports a unidimensional structure. In the current study, internal reliability was good, with a Cronbach’s α of 0.79.

### 2.3. Statistical Analysis

After calculating descriptive statistics (mean and standard deviation), data normality was assessed using skewness, kurtosis, and the Shapiro–Wilk test. While the dependent variables SHALOM Ideal, SHALOM Experienced, and WBI-5 were normally distributed across the weekly frequency of yoga components, normality was violated for all other variables. The independent variable was the frequency of yoga practice, categorized into four groups: once per week, 2–3 times per week, 4–5 times per week, and daily. The yoga frequency variable was assessed separately for each yoga component, and the groupings were mutually exclusive. To examine differences between groups based on weekly yoga practice frequency, either one-way analysis of covariance (ANCOVA) or nonparametric Kruskal–Wallis H tests were applied, depending on the distributional properties of the data. Dependent variables included the SHALOM general factor (Ideal and Experimental), the Personal, Social, Environmental, and Transcendental domains, as well as WBI-5 scores. Post hoc comparisons between yoga frequency groups were conducted using the Mann–Whitney U test with Bonferroni correction (0.05/4), resulting in an adjusted significance threshold of α = 0.0125 [74]. Associations between scale variables were explored using Kendall’s tau correlation. Effect sizes for ANCOVA and Kruskal–Wallis H tests were calculated using partial η^2^ [75], while Cohen’s d was used to estimate effect sizes for Mann–Whitney U tests [76]. Interpretation thresholds for partial η^2^ were: 0.01 (small), 0.06 (medium), and 0.14 (large). Cohen’s d was interpreted as small (<0.3), medium (0.3–0.5), and large (≥0.5) [77]. Age and place of residence, as sociodemographic variables, were found to be associated with the SHALOM and WBI-5 outcomes and were therefore included as covariates in the ANCOVA model to control for potential confounding effects. In contrast, no statistically significant differences were observed between yoga frequency groups for other sociodemographic variables, such as highest educational level and marital status; thus, these variables were not included as grouping factors in subsequent analyses. This observational, cross-sectional study allows identification of associations and correlations but does not support causal inferences, even when considering weekly yoga practice frequency.

All statistical analyses were performed using IBM SPSS Statistics (Version 30.0). The overall significance level was set at α = 0.05. Results with *p* < 0.05 were considered statistically significant, except for Bonferroni-adjusted post hoc comparisons, where the significance threshold was set at α = 0.0125.

## 3. Results

### 3.1. Yoga Practice Characteristics

Based on the data from all 335 participants in the sample, all four yoga components were represented in the sample, with participants reporting weekly practice of āsana, prāṇāyāma, meditation, and relaxation at frequencies ranging from once a week to daily (Table 1).

### 3.2. Associations Between Weekly Frequency of Yoga Practice and Levels of Spiritual- and Subjective Well-Being

Skewness and kurtosis values were examined to assess the normality of the dependent variables across groups, and all three variables met the assumptions of normal distribution. Additionally, Levene’s test for homogeneity of variances indicated that the assumption of equal variances was satisfied (>0.05) for each variable. Using a one-way analysis of covariance (ANCOVA), we examined the main effects of yoga practice frequency (categorized by weekly sessions) on the outcome variables: SHALOM Ideal, SHALOM Experimental, and WBI-5, while controlling for age and place of residence (Table 2).

Higher weekly engagement in all four yoga components; āsana, prāṇāyāma, relaxation, and meditation, was significantly associated with higher levels of both dimensions of SpWB (*p* < 0.001) and SuWB (*p* < 0.05), supporting H1. Daily practice yielded the highest SpWB and SuWB scores, with medium effect sizes observed for SpWB and smaller effects for SuWB, indicating that more frequent yoga participation particularly enhances spiritual well-being while also contributing positively, though to a lesser extent, to overall subjective well-being.

Based on Bonferroni pairwise comparisons, several significant differences were observed between frequency groups across all four yoga components and the three dependent variables. Notably, significant pairwise differences consistently emerged between participants practicing once per week and those practicing daily, as well as, in many cases, between participants practicing 2–3 times per week and those practicing daily. The highest number of pairwise differences for each yoga component was found in relation to the Transcendental subscale of SHALOM.

Assessment of skewness and kurtosis indicated that the distributions of the dependent variables, stratified by weekly yoga practice frequency, deviated from normality. Therefore, nonparametric tests were applied for further statistical analysis (Table 3).

According to the results of the Kruskal–Wallis H test, all four components of yoga were significantly (*p* < 0.05) associated with higher scores on the Personal and Transcendental dimensions of SpWB. Effect sizes for these dimensions were generally small, except for medium effects on the Transcendental dimension for relaxation and meditation. For the Communal dimension, significant (*p* < 0.05) main effects were observed for yoga postures, breathing exercises, and relaxation techniques, all with small effect sizes. In contrast, regarding the Environmental dimension, only yoga postures yielded a significant (*p* < 0.05) main effect, with a small effect size. Overall, these findings indicate that H1 was partially supported, yet in the majority of cases, positive associations were observed, highlighting that frequent yoga practice generally promotes spiritual well-being across multiple dimensions (Table 3).

Mann–Whitney U post hoc analyses revealed significant (*p* < 0.05) pairwise differences between once-per-week and daily practitioners for all variables showing overall group effects in the Kruskal–Wallis H test. Significant differences were also observed in approximately half of the comparisons between 2 and 3 times-per-week practitioners and daily practitioners, as well as between once-per-week practitioners and those practicing more frequently (excluding daily). No significant differences emerged between the 2–3 and 4–5 times-per-week groups. For the Transcendental dimension of spiritual well-being, three significant pairwise differences were found: participants practicing once, 2–3, and 4–5 times per week all differed from daily practitioners, particularly for āsana, relaxation, and meditation components.

### 3.3. Associations Between Spiritual Well-Being (SHALOM) and Subjective Well-Being (SWB)

A majority of the variables did not show a normal distribution; therefore, Kendall’s correlations were used to examine the relationships among continuous variables, including SuWB, SHALOM (SpWB) and its dimensions, as well as age and the number of years practicing yoga (Table 4).

A positive association (*p* < 0.01) was observed between SuWB and SpWB (SHALOM), aligning with H2. The strongest relationships, of moderate magnitude, were found for the overall SHALOM score and the Personal dimension, while weak positive associations were noted with the Communal, Environmental, and Transcendental dimensions, indicating that H2 was largely, though not uniformly, supported (Table 4).

Age showed weak positive correlations with overall SpWB (*p* < 0.01) and most of its dimensions (*p* < 0.05), except for the Social factor. No meaningful relationship emerged between the number of years practicing yoga and any aspect of SpWB (Table 4).

## 4. Discussion

The present study investigated how the weekly frequency of engaging in key yoga components; including physical postures, breathing techniques, relaxation, and meditation, relates to both spiritual and subjective well-being. SpWB was examined in depth using Fisher’s [44] SHALOM (Spiritual Health and Life Orientation Measure), which conceptualizes spirituality as a general overarching factor encompassing four core dimensions: Personal, Social, Environmental, and Transcendental. The SHALOM instrument provides a comprehensive and inclusive framework suitable for individuals across diverse religious, spiritual, and non-religious orientations. However, considering the nature of the methodology, several limitations should be acknowledged when interpreting these findings. As a cross-sectional study, the design precludes conclusions about causality between yoga practice, spirituality, and well-being.

Consistent with H1, more frequent engagement in āsana, prāṇāyāma, relaxation, and meditation was generally associated with higher levels of the general factor of spiritual well-being (SpWB), with medium effect sizes, and subjective well-being (SuWB), with small effect sizes. Moreover, engaging in at least two or more components of yoga exercise—and in some cases, all components—was positively associated with higher scores in each dimension of spirituality. Daily practice was associated with the greatest scores, with medium effect sizes observed for the Transcendental dimension, particularly for relaxation and meditation, while SuWB showed smaller gains. All four components linked positively, though modestly, to the Personal dimension. In the Communal dimension, āsana, prāṇāyāma, and relaxation demonstrated small positive associations, whereas meditation did not reach significance. For the Environmental dimension, only āsana showed a small positive relationship. Pairwise comparisons further indicated that significant differences were primarily observed between once-per-week and daily practitioners, and in several cases between 2 and 3 times per week and daily practitioners, while no significant differences emerged between intermediate frequency groups.

These findings partially support H1 and indicate that both the frequency and type of yoga practice are associated with higher scores in specific dimensions of spiritual well-being, while also showing component-specific patterns related to subjective well-being. The results suggest that more frequent participation in yoga is related to a stronger sense of connectedness to oneself, to others, to the environment, and to God, and may be linked to more favorable mental health indicators. Enhanced spiritual and subjective well-being were also linked to reduced stress-related negative emotions [31,36], indicating that regular yoga practice may serve as an effective pathway toward improved psychological resilience and emotional balance.

The results of our study are consistent with a German national study by Cramer et al. [78] and a UK survey by Cartwright et al. [79], both recognizing spirituality as a significant driver for engaging in yoga practice. Cramer et al. (2015), including motivational insights from an earlier survey [80], reported that 29.4% of yoga practitioners in Germany named spiritual reasons for practising yoga. The UK survey by Cartwright et al. [79] noted that while initial motivations often centered on general wellness and fitness, nearly half (47%) of participants reported a change in their primary motivations over time, with spirituality becoming important for 21% of current practitioners. This collective evidence highlights that spirituality is increasingly recognized as a meaningful motivation in Western contexts and often evolves into a more holistic psycho-spiritual approach as individuals deepen their yoga practice.

The present findings also demonstrated a consistent positive link between yoga practice and psychological health, as measured by the WHO-5 Well-Being Index (WBI-5). As hypothesized in H1, more frequent weekly practice of āsana, prāṇāyāma, relaxation, and meditation was positively associated with subjective well-being (SuWB), with a small effect size. The strongest relationship emerged with the Personal factor, while weaker but consistent associations were observed across the Social, Environmental, and Transcendental dimensions. These results suggest that the psycho-spiritual benefits of yoga are multifaceted and cumulative across its various practices.

Consistent with H2, overall spiritual well-being (SpWB, SHALOM) was positively correlated with subjective well-being (SuWB). The strongest relationships, of moderate magnitude, were observed for the overall suWB score and the Personal dimension, suggesting that personal aspects of spirituality are particularly relevant to perceived SuWB. Weak positive associations were found between SuWB and the Communal, Environmental, and Transcendental dimensions, indicating that while these dimensions contribute to overall well-being, their impact is less pronounced. Collectively, these findings largely support H2, though the strength of associations varies across dimensions.

The observed correlations align with broader research linking SuWB to greater resilience and reduced risk of depression. Wood and Joseph [81] showed that low SuWB was a strong long-term predictor of depression, while Mangelli et al. [82] observed associations between SuWB and lower depressive symptoms in chronic illness. Yoga, by promoting both SpWB and SuWB, may therefore serve as a holistic tool for mental health [79].

Cartwright et al. [79] further reported that a greater emphasis on spirituality in yoga practice was associated with higher SuWB, supporting our conclusion that spiritual focus within yoga practice linked to greater psychological wellness. Their findings revealed that, although initial motivations often included physical fitness and general wellness, many practitioners later shifted toward more spiritual and self-transcendent goals—illustrating yoga’s potential to promote psycho-spiritual integration over time.

Yoga thus functions as a significant method for mental health, with regular practice positively influencing both SuWB [9,82] and SpWB [1]. Evidence shows that consistent yoga practice—at least twice per week—is positively correlated with key dimensions of psychological well-being such as meaning in life and gratitude [83]. The depth of personal commitment also appears essential, as highly committed practitioners report significantly higher levels of religiousness and SpWB than those with only moderate or marginal involvement in yoga [33,84].

These findings suggest that the psychological benefits of yoga are deeply rooted in its internal, experiential, and spiritual dimensions, extending well beyond its physical components. While regular practice is a prerequisite for realizing potential spiritual benefits, the relationship between spirituality and SuWB appears to be more strongly associated with positive mental health outcomes than objective metrics such as frequency or duration of practice [83]. The spiritual qualities cultivated through yoga; inner peace, hope, faith, and compassion, are directly linked to greater psychological resilience and higher levels of SuWB [33]. The present results support the assumption that spirituality is inherently connected to and nurtured through yoga practice.

Weekly engagement in yoga is associated with higher levels of both SpWB and SuWB, which are interrelated and, in turn, linked with better mental health outcomes. As enhanced mental well-being is inversely related to common affective disorders such as depression, anxiety, and stress [85,86], our findings suggest that yoga may be relevant to the promotion of mental health, but causal and preventive effects require confirmation in longitudinal and experimental studies.

Additional analyses indicated that age was weakly positively correlated with overall SpWB and most dimensions, except the Social factor, while years of yoga practice showed no meaningful associations with any SpWB aspect, suggesting that current engagement, rather than long-term practice, may be more relevant for spiritual well-being.

Several limitations should be acknowledged when interpreting these findings. Data collection relied on self-report questionnaires, which may introduce response bias. As a cross-sectional study, the design precludes conclusions about causality between yoga practice, spirituality, and well-being. The sample consisted exclusively of current, self-selected yoga practitioners, potentially leading to an inherent positivity bias regarding yoga’s benefits. Although the likelihood was considered low, the possibility of multiple submissions by the same participant cannot be entirely excluded in this online, Google Forms-based cross-sectional survey, which may have affected the internal validity of the findings. The lack of a control group also limits the strength of comparative conclusions. Furthermore, the sample was demographically unbalanced, with a predominance of women (88.1%) and a high proportion of participants with tertiary education (69.0% university degree, 3.0% PhD), possibly indicating higher health consciousness and reducing the generalizability of results.

Future research should employ longitudinal and controlled designs to explore causal mechanisms underlying the observed associations. Furthermore, a mixed-method study design could enhance the understanding of the relationships among the examined variables by combining self-report questionnaires with objective biological markers, such as physiological and neurobiological factors, including indicators of inflammation (CRP, interleukins) and stress response (cortisol), thereby helping to reduce response bias. Comparisons across different yoga styles and religious affiliations may provide deeper insights into how specific practices contribute to spiritual and psychological well-being. In addition, future studies would benefit from a more balanced distribution of gender, educational level, and other sociodemographic characteristics in the study sample.

## 5. Conclusions

Weekly yoga practice showed modest associations with both spiritual well-being (SpWB) and subjective well-being (SuWB), particularly in the Personal and Transcendental dimensions of spirituality. All measured yoga components—āsanas, prāṇāyāma, relaxation, and meditation—were generally positively associated with SpWB and SuWB, though effect sizes were small to moderate. Moderate correlations between SpWB and SuWB suggest a tentative link between spirituality and mental health. This finding is of potential public health relevance, as higher SuWB may be associated with lower levels of distress-related symptoms, highlighting the importance of considering spiritual dimensions in mental well-being interventions. Overall, these findings point to potential benefits of regular yoga practice for personal and spiritual development but should be interpreted cautiously and warrant further investigation.

## Figures and Tables

**Table 1:** Frequency of participation in yoga components (*n* = 335).

Frequency	Asana	Prāṇāyāma	Relaxation	Meditation
once a week	57 (17%)	91 (27.2%)	69 (20.6%)	126 (37.6%)
2–3 times a week	121 (36.1%)	118 (35.2%)	118 (35.2%)	96 (28.8%)
4–5 times a week	82 (24.5%)	56 (16.7%)	76 (22.7%)	38 (11.3%)
daily	75 (22.4%)	70 (20.9%)	72 (21.5%)	75 (22.4%)
Total	335 (100%)	335 (100%)	335 (100%)	335 (100%)

Note: Asana = yoga postures; Prāṇāyāma = breathing techniques. The sample contains no missing data.

**Table 2:** One-Way Analyses of Covariance (ANCOVA) in SpWB and SuWB according to yoga components weekly frequency groups (*n* = 335).

Measure	Once a Week		2–3× a Week		4–5× a Week		Daily		F	*p*	η^2^_p_
	M	SD	M	SD	M	SD	M	SD			
Asana	*n* = 57		*n* = 121		*n* = 82		*n* = 75				
SH-I	79.47	13.39	81.11	12.73	84.82	10.86	88.60	9.63	9.64	<0.001	0.081
SH-E	72.16	13.38	75.32	14.01	76.21	13.16	82.52	11.27	8.17	<0.001	0.069
WBI-5	9.32	2.57	9.93	2.55	9.85	2.60	10.75	2.58	3.63	0.013	0.032
Prāṇāyāma	*n* = 91		*n* = 118		*n* = 56		*n* = 70				
SH-I	80.41	12.62	81.89	12.84	84.57	10.48	88.97	9.79	7.58	<0.001	0.065
SH-E	72.64	13.90	75.80	13.36	76.68	12.78	83.10	11.57	8.21	<0.001	0.070
WBI-5	9.30	2.71	9.99	2.54	10.04	2.50	10.84	2.29	4.64	0.003	0.041
Relaxation	*n* = 69		*n* = 118		*n* = 76		*n* = 72				
SH-I	78.62	13.22	81.19	12.66	85.83	10.19	89.10	9.37	12.78	<0.001	0.104
SH-E	71.61	15.24	74.86	12.96	78.13	11.65	82.67	12.13	10.06	<0.001	0.084
WBI-5	9.35	2.87	9.70	2.52	10.43	2.53	10.60	2.25	4.21	0.006	0.037
Meditation	*n* = 126		*n* =96		*n* = 38		*n* = 75				
SH-I	79.68	13.47	82.81	10.84	87.16	9.32	88.56	10.56	10.58	<0.001	0.088
SH-E	72.52	14.36	77.69	12.09	78.89	12.73	80.96	12.45	6.99	<0.001	0.060
WBI-5	9.39	2.87	10.24	2.29	10.53	2.42	10.40	2.33	3.71	0.012	0.033

Note. M = mean; SD = standard deviation; SH-I = SHALOM Ideal; SH-E = SHALOM Experimental; covariates appearing in the model are evaluated at the following values: age = 47.21, living place = 2.45.

**Table 3:** Kruskal–Wallis H tests results in SpWB and SuWB according to yoga components weekly frequency groups (*n* = 335).

Measure	Once a Week		2–3× a Week		4–5× a Week		Daily		χ^2^(3)	*p*	η^2^
	M	SD	M	SD	M	SD	M	SD			
Asana	*n* = 57		*n* = 121		*n* = 82		*n* = 75				
SH-P	19.75	3.43	20.03	3.72	20.15	3.46	21.33	3.15	9.22	0.026	0.019
SH-C	18.32	3.45	19.19	3.44	19.62	2.83	20.76	2.95	19.80	<0.001	0.051
SH-E	19.07	4.11	20.27	3.76	19.67	3.77	21.08	3.40	10.31	0.016	0.022
SH-T	15.02	6.44	15.83	6.60	16.77	6.13	19.35	5.05	18.23	<0.001	0.046
Prāṇāyāma	*n* = 91		*n* = 118		*n* = 56		*n* = 70				
SH-P	19.43	3.96	20.10	3.43	20.34	3.21	21.76	2.81	16.73	<0.001	0.041
SH-C	19.08	3.26	19.08	3.50	19.59	2.88	20.67	2.98	11.43	0.010	0.025
SH-E	19.47	3.83	20.25	3.65	19.77	3.99	20.93	3.72	7.03	NS	0.012
SH-T	14.66	6.59	16.36	6.23	16.98	5.92	19.74	5.15	26.71	<0.001	0.072
Relaxation	*n* = 69		*n* = 118		*n* = 76		*n* = 72				
SH-P	19.46	4.07	19.93	3.43	20.82	3.00	21.18	3.36	9.98	0.019	0.021
SH-C	19.04	3.35	18.97	3.38	19.72	2.98	20.56	3.21	11.37	0.010	0.025
SH-E	19.21	4.22	19.98	3.72	20.29	3.29	20.94	3.85	7.65	NS	0.014
SH-T	13.88	6.85	15.97	6.34	17.30	5.65	19.99	4.70	33.12	<0.001	0.091
Meditation	*n* = 126		*n* =96		*n* = 38		*n* = 75				
SH-P	19.39	3.88	20.82	3.04	20.71	3.21	20.97	3.31	11.74	0.008	0.026
SH-C	18.92	3.52	19.50	3.15	19.92	3.16	20.25	2.93	6.88	NS	0.012
SH-E	19.67	4.00	20.46	3.36	20.37	3.71	20.24	4.00	2.18	NS	0.002
SH-T	14.54	6.61	16.91	6.04	17.90	5.86	19.49	4.97	29.48	<0.001	0.080

Note. *n* = 335; M = mean; SD = standard deviation; SH-P = SHALOM Personal; SH-C = SHALOM Communal; SH-E = SHALOM Environmental; SH-T = SHALOM Transcendental; NS = not significant.

**Table 4:** Kendall correlations between SpWB and SuWB (*n* = 335).

	SH-P	SH-C	SH-E	SH-T	SHALOM
WBI-5	0.35 **	0.27 **	0.29 **	0.26 **	0.34 **
Age	0.09 *	0.07	0.08 *	0.08 *	0.10 **
Yoga time	0.00	0.01	−0.03	0.01	0.00

Note. SpWB = Spiritual well-being; SuWB = Subjective well-being; WBI-5 = WHO Well-being Index shortened form; SH-P = SHALOM Personal; SH-C = SHALOM Communal, SH-E = SHALOM Environmental, SH-T = SHALOM Transcendental; SHALOM = Spiritual Health and Life Orientation Measure. * *p* < 0.05, ** *p* < 0.01.

## Data Availability

All data are included in the study.

## Abbreviations

The following abbreviations are used in this manuscript:

ANCOVA	Analysis of Covariance
PMH	Positive Mental Health
SHALOM	Spiritual Health and Life-Orientation Measure
SpWB	Spiritual Well-being
SuWB	Subjective Well-being
WBI-5	WHO-5 Well-Being Index
